# *Toxoplasma gondii* GRA8-derived peptide immunotherapy improves tumor targeting of colorectal cancer

**DOI:** 10.18632/oncotarget.27417

**Published:** 2020-01-07

**Authors:** Jae-Sung Kim, Daeun Lee, Donggyu Kim, Seok-Jun Mun, Euni Cho, Wooic Son, Chul-Su Yang

**Affiliations:** ^1^Department of Molecular and Life Science, Hanyang University, Ansan 15588, S. Korea; ^2^Department of Bionano Technology, Hanyang University, Seoul 04673, S. Korea

**Keywords:** *Toxoplasma gondii* GRA8 peptide, mitochondria, tumor-targeting, metabolism

## Abstract

Targeted tumor and efficient, specific biological drug delivery *in vivo* has been one of the main challenges in protein-based cancer-targeted therapies. Mitochondria are potential therapeutic targets for various anti-cancer drugs. We have previously reported that protein kinase Cα-mediated phosphorylation of *Toxoplasma gondii* GRA8 is required for mitochondrial trafficking and regulating the interaction of the C-terminal of GRA8 with ATP5A1/SIRT3 in mitochondria. Furthermore, SIRT3 facilitates ATP5A1 deacetylation, mitochondrial activation, and subsequent antiseptic activity *in vivo*. Herein we developed a recombinant acidity-triggered rational membrane (ATRAM)-conjugated multifunctional GRA8 peptide (rATRAM-G8-M/AS) comprising ATRAM as the cancer-targeting cell-penetrating peptide, and essential/minimal residues for mitochondrial targeting or ATP5A1/SIRT3 binding. This peptide construct showed considerably improved potency about cancer cell death via mitochondria activity and biogenesis compared with rGRA8 alone in HCT116 human carcinoma cells, reaching an IC_50_ value of up to 200-fold lower *in vitro* and 500-fold lower *in vivo*. Notably, rATRAM-G8-M/AS treatment showed significant therapeutic effects in a mouse xenograft model through mitochondrial metabolic resuscitation, and it produced negligible immunogenicity and immune responses *in vivo*. Thus, these results demonstrate that rATRAM-G8-M/AS represents a useful therapeutic strategy against tumors, particularly colon cancer. This strategy represents an urgently needed paradigm shift for therapeutic intervention.

## INTRODUCTION

Cancer is the primary cause of mortality worldwide, and in the United States cancer related deaths are second only to heart disease related mortality [1]. Metabolism is important not only in cancer development but also in its prevention [2, 3]. Cancer cells have high rates of metabolism and require adenosine triphosphate (ATP) for their survival, proliferation and growth. ATP production in mitochondria is regulated acetylation and deacetylation of many mitochondrial enzymes and NAD-dependent protein deacetylase sirtuin-3 (SIRT3) present in mitochondria plays an important role in this process [4–6]. Therefore, deciphering how the metabolic regulators in cancer cells are specifically changed would help in the development of more effective anti-cancer therapies. Therapeutic approaches that target mitochondria are considered to provide novel means to combat cancer and to reduce associated mortality and even to reverse the oncogenic process. Such metabolic intervening measures, called “metabolic resuscitation”, employ either nutritional or pharmacological agents to improve mitochondrial function [2, 4, 7, 8]. However, the underlying mechanisms for the metabolic resuscitation induced by the dense granule protein GRA8 of *Toxoplasma gondii* (*T. gondii*) in tumor cells are yet to be investigated [8].

We previously discovered that protein kinase Cα (PKCα)-phosphorylated *T. gondii* GRA8 is transferred to the mitochondria and interacts with mitochondrial SIRT3. SIRT3 undergoes deacetylation with ATP synthase F1 subunit alpha (ATP5A1) and regulates mitochondrial activity to contribute to antiseptic activities *in vivo* [8, 9].

ATP5A1 codes for one of the subunits of ATP synthase complex in mitochondrial membrane. During cellular respiration, an electrochemical gradient of protons across the mitochondrial inner membrane is generated due to the activity of electron transport chain. Coupling of this electrochemical gradient across the inner membrane with the mitochondrial ATP synthase activity drives ATP synthesis, a process called during oxidative phosphorylation (OXPHOS). Notably, acetylation status of many mitochondrial proteins controls the synthesis of ATP. Thus, deacetylation of the ATP5A1 subunit of ATP synthase complex directly enhances ATP synthesis, and indirectly affects ATP production by decreasing the flux through fatty acid oxidation pathway [10–13]. In the absence of SIRT3, mitochondrial proteins become hyperacetylated, have reduced activity, and lead to mitochondrial dysfunction [10]. Deacetylated ATP5A1 is involved in several mitochondrial functions, and SIRT3 is able to orchestrate the overall alterations in mitochondrial function, that is necessary for the growth of cancers. SIRT3 can act like a tumor suppressor as well as an oncogene, and thus modulate cell death by affecting the main regulatory factors and their signaling pathways in cancer [5, 6, 14, 15]. Thus, the GRA8-mediated cellular mechanisms that regulate mitochondrial metabolism could be exploited in therapeutic approaches against tumors similar to strategies that have been used against sepsis [2, 8, 16].

Proteins and peptides have significant application as biological therapeutics. Presently, small-molecule drugs comprise majority of the pharmaceutical market; unlike these commonly used small-molecule drugs, the naturally occurring or derived proteins and peptides can have significantly better selectivity because their ability to interact with their corresponding targets is dependent on several points of contact [17, 18]. In addition, enhanced selectivity has the potential to lower toxicity and side effects. Inasmuch as it is possible to design peptides to affect a wide range of targets, there are numerous possibilities of applying peptide therapeutics in several fields including endocrinology, immunology, oncology, and infectious disease [8, 18, 19]. In this study, we built upon the discoveries of the minimal peptide moieties for mitochondrial targeting and ATP5A1/SIRT3 binding, previously reported to be important in therapeutic approaches [8].

Despite significant progress in the therapeutic development of peptides and proteins, measures to improve their systemic stability as well as site-specific delivery still need to be worked-out. Furthermore, one of the main obstacles in the clinical development of cell-penetrating peptides is their lack of target specificity [18, 20, 21]. To examine this problem, we investigated the developed strategies using GRA8 peptides that are coupled with cancer-targeting peptides (CTPs). The tumor microenvironment has unique characteristics, that are used by the CTPs for improving cancer cell targeting specificity.

We investigated the multifunctional GRA8 peptide for mitochondrial metabolic reprogramming and its conjugation with the acidity-triggered rational membrane (ATRAM) to provide a better and effective strategy for delivering the anti-cancer therapeutics. Notably [1], Design and expression of ATRAM-conjugated multifunctional GRA8 peptide to target tumor cells *in vitro* and *in vivo* [2], rATRAM-GRA8-M/AS induced HCT116 cell death by mitochondrial activation [3], rATRAM-GRA8-M/AS showed antitumor activity in HCT116 xenografts.

## RESULTS

### rGRA8-induces colon cell death via PKCα-SIRT3-ATP5A1 pathways

**Figure 1:**
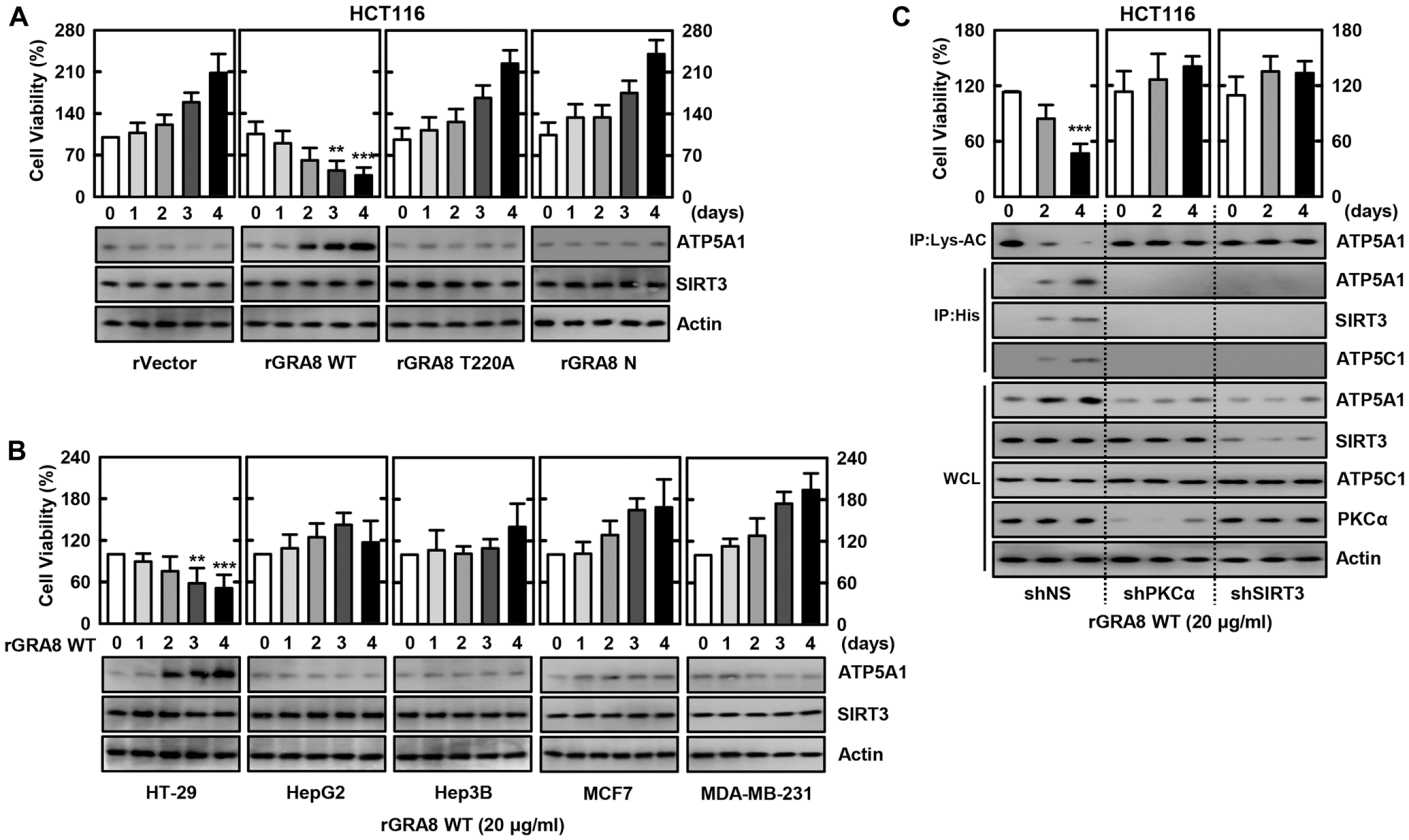
The rGRA8 treatment increases the HCT116 cell death by mitochondria pathways. (**A** and **B**) The various cancer cells were incubated with WT rGRA8 (20 μg/ml) and its mutants for the indicated times and then cell viability measured with MTT assay (upper) and subjected to IB with αATP5A1, αSIRT3, or αActin (lower). (**C**) HCT116 cells were transduced with lentivirus-shRNA-NS or lentivirus-shRNA-PKCα or SIRT3 (MOI = 100) with polybrene (8 μg/mL) (right) for 2 days. The cells were incubated with WT rGRA8 for the indicated times and subjected to IP with αLys-AC or αHis and IB with αATP5A1, αATP5C1, αSIRT3, αPGC-1, αPKCα, or αActin. The data are representative of five independent experiments with similar results. Statistical significance was determined by Student’s *t*-test with Bonferroni adjustment; ^**^ *P* < 0.01, ^***^ *P* < 0.001 compared with rVector-treated.

PKCα-phosphorylated GRA8 associates with mitochondrial SIRT3; SIRT3 associates with ATP5A1 and controls its activity by affecting acetylation status [8, 9]. Deacetylated ATP5A1 participates in several mitochondrial functions, and SIRT3 can orchestrate the overall changes in mitochondrial function, that are critical for cancer growth [14, 22]. We determined whether rGRA8 or its mutants caused cell death by MTT assay in various cancer cell lines. Figure 1A and 1B show that WT rGRA8 caused cell death in human colon-cancer cell lines (HCT116 and HT-29) in a time-dependent manner, but rGRA8 mutants (rGRA8 T220A mutant lost both PKCα-mediated GRA8 phosphorylation and mitochondrial trafficking; rGRA8 N mutant (aa 1-241) lost GRA8-SIRT3/ATP5A1 interaction but retained the both PKCα-mediated phosphorylation and mitochondrial trafficking) failed to do so. Similar to the treatment of macrophages [8], the treatment of HCT116 cells with WT rGRA8 significantly elevated the interaction of rGRA8 to mitochondrial SIRT3, ATP5A1 and ATP5C1 as well as increased ATP5A1 deacetylation by immunoprecipitation (IP) and immunoblot (IB) (Figure 1C). However, in the PKCα or SIRT3-deficiency cells, GRA8 binding with those proteins and acetylation patterns disappeared using shRNA lentiviral transduction. Therefore, WT rGRA8 induces cell death in human colon-cancer cells via mitochondrial metabolic resuscitation pathways.

### ATRAM-conjugated GRA8 peptide designed to target tumors

**Figure 2:**
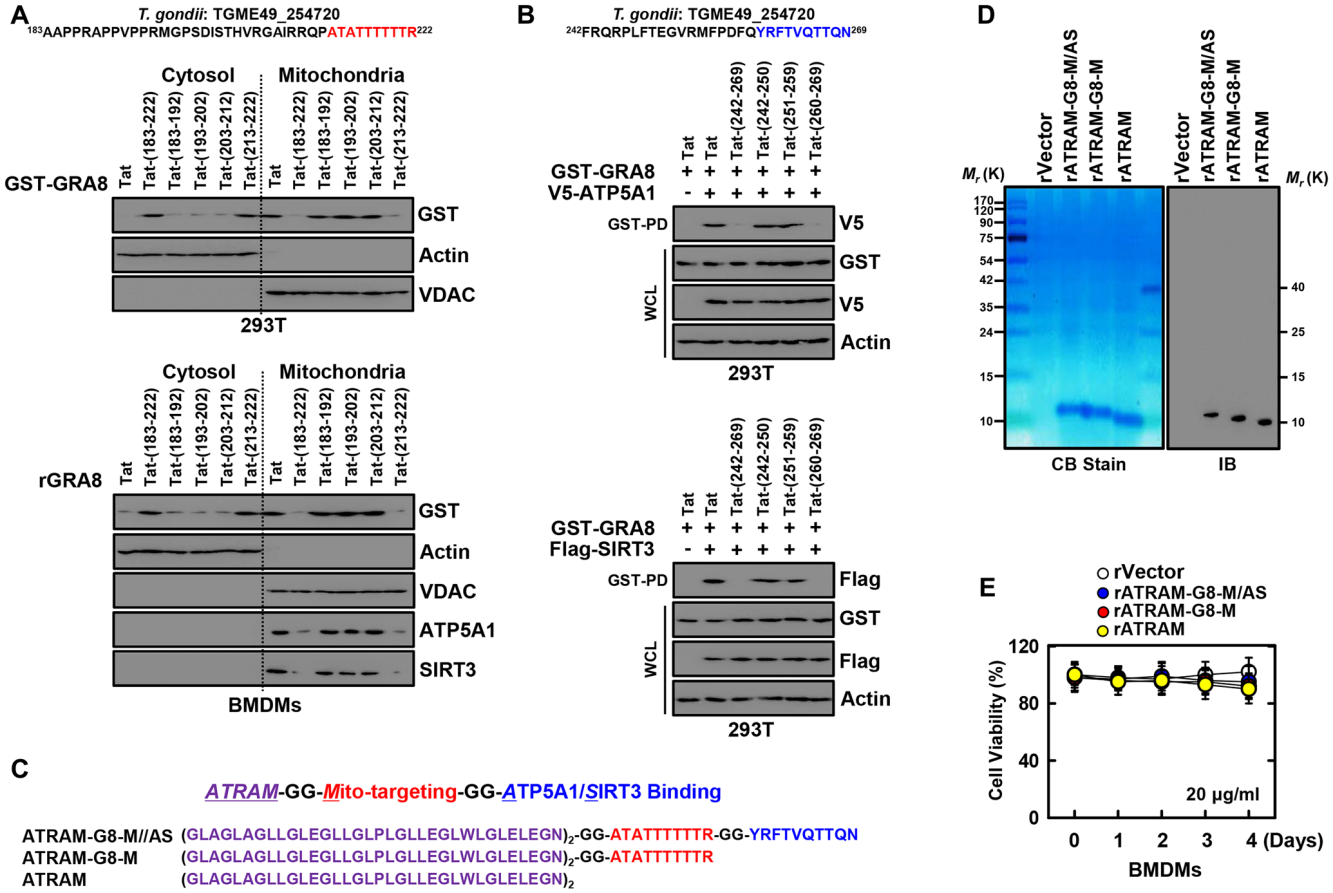
Design and expression of ATRAM-conjugated multifunctional GRA8 peptide-based protein. (**A**) Subcellular fractionation of 293T cells are expressing GST-GRA8 WT or incubation with WT rGRA8 (1 μg/ml) BMDMs and treated with several Tat-GRA8 peptide (10 µM) for 6 h. Mitochondrial and cytosolic fractions were fractionated and analyzed for expression of GST by IB. Purity of the fractions was assessed by blotting for VDAC (mitochondrial protein) and Actin (cytosolic protein). (**B**) At 12 hr post-transfection with mammalian GST-GRA8 constructs together with V5-ATP5A1, or Flag-SIRT3 and 293T cells treated with several Tat-GRA8 peptide (10 µM) for 6 h. 293T cells were used for GST pulldown, followed by IB with αV5 or αFlag. WCLs were used for IB with αGST, αV5, αFlag or αActin. (**C**) Schematic in design of ATRAM-GRA8-M/AS and its mutants. (**D**) Bacterially purified 6xHis-rATRAM-GRA8-M/AS and its mutants were analyzed by Coomassie blue staining (left) or immuno blotting (IB) with αHis (right). (**E**) BMDMs were incubated with rATRAM-GRA8-M/AS and its mutants (20 μg/ml) for the indicated times and then cell viability measured with MTT assay. The data are representative of four independent experiments with similar results (A, B, D, and E).

Our earlier work on interaction of GRA8 revealed that the 40-amino acid sequence (aa 183–222) or 28-amino acid sequence (aa 242–269) at the C-terminus of GRA8 is sufficient for mitochondrial targeting or ATP5A1/SIRT3 interaction, respectively [8]. Thus, the transduction-domain of HIV-1 Tat protein was added to the 9–10-amino acid sequence of GRA8 as a retro-inverso peptide, known as the Tat-GRA8 peptide, for intracellular delivery to prevent proteolytic breakdown [22, 23] (Tables 1 and 2). These peptides were tested for their potential minimal residues of GRA8 for mitochondrial targeting and ATP5A1/SIRT3 interaction in 293T cells. At 12-h post-transfection with GST-GRA8 or treatment with rGRA8, 293T cells or bone marrow derived macrophages were incubated with Tat alone or different Tat-GRA8 peptides followed by subcellular fractionation to determine the mitochondrial localization of GRA8. This showed that the Tat-GRA8 (aa 213–222) peptide was capable of effectively blocking mitochondrial targeting within the incubation period of 6 hours, while the Tat peptide *per se* was unable to do so (Figure 2A). Furthermore, the Tat-GRA8 (aa 260–269) peptide was able to efficiently block the GRA8-ATP5A1/SIRT3 interaction within 6 h of incubation as determined by GST-pull-down assay in 293T cells (Figure 2B and Supplementary Figure 1). Thus, these data revealed the minimal region on GRA8 for mitochondria targeting (aa 213–222) and ATP5A1/SIRT3 binding (aa 260–269).

**Table 1:** Amino acid sequences of *T. gondii* GRA8

Gene	Accession no	Sequence
TGME49_254720	XP_002369526.1	MALPLRVSATVFVVFAVFGVARAMNGPLSYHPSSYGASYP NPSNPLHGMPKPENPVRPPPPGFHPSVIPNPPYPLGTPAGMP QPEVPPLQHPPPTGSPPAAAPQPPYPVGTPGMPQPEIPPVHR PPPPGFRPEVAPVPPYPVGTPTGMPQPEIPAVHHPFPYVTTTT TAAPRVLVYKIPYGGAAPPRAPPVPPRMGPSDISTHVRGAIR RQP**ATATTTTTTR**NVLLRTAILAAAAATLIALFRQRPLFTEG VRMFPDFQ**YRFTVQTTQN**

**Table 2:** Sequences of *T. gondii* GRA8 peptide

Name	Sequence (From N to C)
Tat	RRRQRRKKRGY
Tat-GRA8-(183-222)	RRRQRRKKRGY-G-RTTTTTTATAPQRRIAGRVHTSIDSPGMRPPVPPARPPAA
Tat-GRA8-(183-192)	RRRQRRKKRGY-G-PVPPARPPAA
Tat-GRA8-(193-202)	RRRQRRKKRGY-G-TSIDSPGMRP
Tat-GRA8-(203-212)	RRRQRRKKRGY-G-PQRRIAGRVH
Tat-GRA8-(213-222)	RRRQRRKKRGY-G-RTTTTTTATA
Tat-GRA8-(242-269)	RRRQRRKKRGY-G-NQTTQVTFRYQFDPFMRVGETFLPRQRF
Tat-GRA8-(242-250)	RRRQRRKKRGY-G-ETFLPRQRF
Tat-GRA8-(251-259)	RRRQRRKKRGY-G-QFDPFMRVG
Tat-GRA8-(260-269)	RRRQRRKKRGY-G-NQTTQVTFRY

The ATRAM showed a strong binding to cancer cells in acidic environment, thereby underscoring its superior CTPs [20, 21]. Thus, we designed the ATRAM-conjugated multifunctional GRA8 peptide for targeting tumors and activating mitochondrial metabolism (Figure 2C). To assess the function of the ATRAM-conjugated multifunctional GRA8 peptide in cancer cells, we produced His-tagged rATRAM-GRA8-M/AS and its mutants in bacteria and purified by affinity chromatography as previously described [8, 19]. The purified rATRAM-GRA8-M/AS (10 kDa) was confirmed by SDS-PAGE and immunoblotting (Figure 2D). No significant cytotoxic differences in macrophages were observed for rATRAM-GRA8-M/AS or its mutants compared with the vector controls in BMDMs using MTT assay (Figure 2E). Therefore, the ATRAM-conjugated multifunctional GRA8 peptide is expected to be specific for cancer cells by regulating cancer cell mitochondrial metabolism.

### rATRAM-GRA8-M/AS shows mitochondrial activity and biogenesis in HCT116

**Figure 3:**
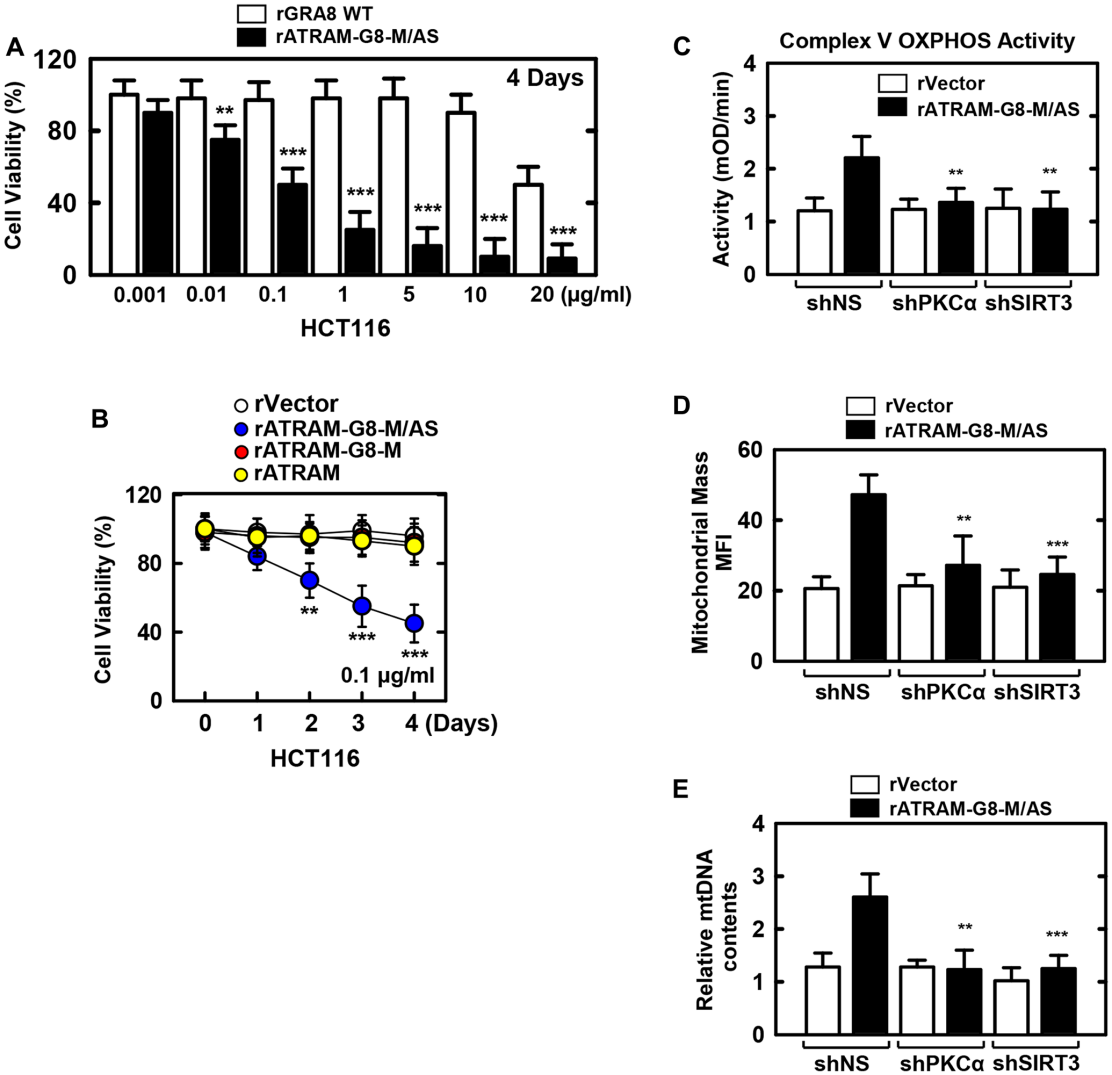
The rATRAM-GRA8-M/AS-induced the HCT116 cell death by mitochondria activation. (**A** and **B**) HCT116 cells were incubated with WT rGRA8 or rATRAM-GRA8-M/AS and its mutants for the indicated times and then cell viability measured with MTT assay. (**C–E**) HCT116 cells were transduced with lentivirus-shRNA-NS or lentivirus-shRNA-PKCα or SIRT3 (MOI = 100) with polybrene (8 μg/mL) (right) for 2 days. The cells were stimulated with rATRAM-GRA8-M/AS for 1 day and subjected to enzymatic activity of OXPHOS V (C), Mitotracker fluorescence signals assessed by a flow cytometric analysis. Bar graph indicates the mitochondrial mass MFIs (D), or mtDNA content in BMDMs measured by quantitative real-time PCR. The mtDNA content was normalized to nuclear DNA (E). Statistical significance was determined by Student’s *t*-test with Bonferroni adjustment; ^**^ *P* < 0.01, ^***^ *P* < 0.001 compared with control-treated. The data are representative of five independent experiments with similar results.

We verified whether rATRAM-GRA8-M/AS had rGRA8-mimetic pharmacological and biological profiles [8]. Consistent with the activity of rGRA8 alone, rATRAM-GRA8-M/AS also caused cell death in HCT116 cells in a dose-dependent manner. Remarkably, rATRAM-GRA8-M/AS had an IC_50_ of 0.1 μg/ml, which was a 200-fold improvement in IC_50_ compared with that of 20 μg/ml found for rGRA8 alone (Figure 3A). Furthermore, no significant differences in cytotoxicity were observed between mutants rATRAM-GRA8-M (lost ATP5A1/SIRT3 binding but retained mitochondrial targeting) or rATRAM (lost both mitochondrial targeting and ATP5A1/SIRT3 binding) and vector controls in HCT116 cells (Figure 3B). This finding indicates that GRA8 regions for both mitochondrial targeting and ATP5A1/SIRT3 are essential for cancer cell death.

Thereafter, we examined the mitochondrial metabolism parameters of rATRAM-GRA8-M/AS. As shown in Figure 3C–3E, rATRAM-GRA8-M/AS enhanced the complex V OXPHOS activity by MitoTox complex V OXPHOS activity assay kit, mitochondrial mass by TMRE-mitochondrial membrane potential analysis by flow cytometry, and mtDNA/nDMA ratio-dependent mitochondrial DNA content by PCR analysis via SIRT3 and PKCα in HCT116 cells. Thus, rATRAM-GRA8-M/AS acts as a selective and potent metabolic-modulatory agent by mitochondria-targeted metabolic resuscitation.

### Antitumor activity of rATRAM-GRA8-M/AS in HCT116 xenografts

We examined the pharmacokinetics and pharmacodistribution of rATRAM-GRA8-M/AS before assessing its therapeutic potential *in vivo*. rATRAM-GRA8-M/AS proteins were localized in tumor cells in several organs as detected by IB. This localization was sustained for up to 2 days and slowly dissipated by the 3rd day (Supplementary Figure 2A).

**Figure 4:**
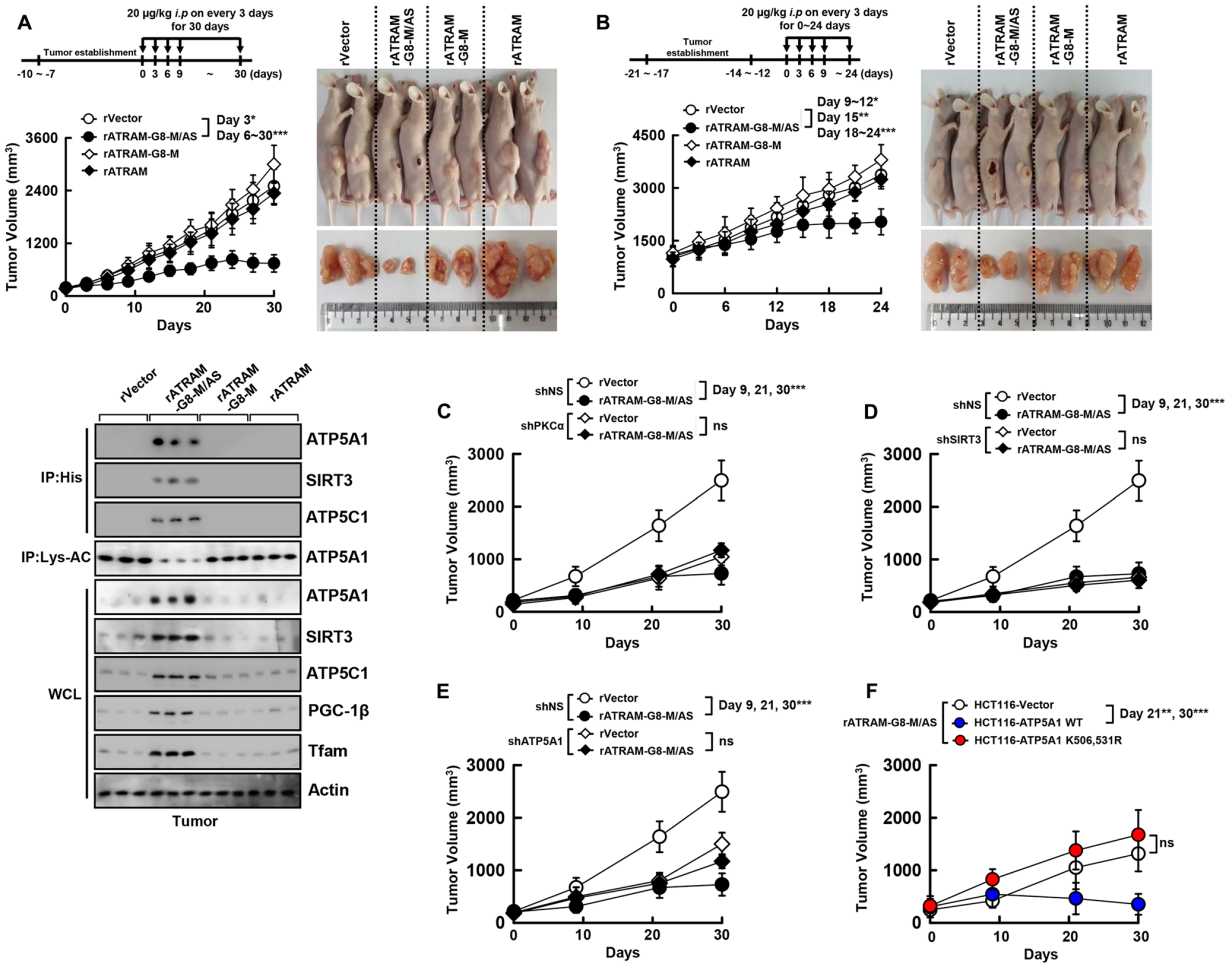
The rATRAM-GRA8-M/AS showed an antitumor activity in HCT116 xenografts. (**A** and **B**) Schematic of the xenograft model treated with or rATRAM-GRA8-M/AS or its mutants (upper). HCT116 cells were subcutaneously injected into the flanks of nude mice. The length and width of the tumors were measured using calipers, and the tumor volume was calculated every third day for 30 days. At day 15 after administration, the mice were euthanatized, and the interaction with GRA8, levels of acetylation of ATP5A1, or expression of OXPHOS were analyzed (A, lower). Representative images of tumors from mice treated with rATRAM-GRA8-M/AS or mutants on day 15 (right). (**C–F**) HCT116 cells were transduced with lentivirus-shRNA-NS or lentivirus-shRNA-PKCα, SIRT3, or ATP5A1 with polybrene (C–E) or HCT116-expressed ATP5A1 WT and mutant cells were subcutaneously injected into the flanks of nude mice. Individual tumor volumes from each mouse from each group were averaged and plotted against the number days postinoculation. Statistical significance was determined by two-way analysis of variance (ANOVA) with Tukey’s posttest; ^*^ *P* < 0.05, ^**^ *P* < 0.01, ^***^ *P* < 0.001 compared with rVector. Each group contained ten mice. The data are representative of two independent experiments with similar results.

We evaluated the antitumor activity of rATRAM-GRA8-M/AS in xenograft mice containing HCT116 cells. rATRAM-GRA8-M/AS (20 µg/kg, intraperitoneal injection) treatment inhibited the growth of tumors in mice initially with growing tumors (Figure 4A) and actively growing tumors (Figure 4B). In addition, we tested whether rATRAM-GRA8-M/AS was pharmacologically effective *in vivo* by determining the profiles of mitochondrial target protein-binding and ATP synthesis activity to assess its potential clinical applicability. Consistent with the CLP-induced sepsis model [8], treatment with rATRAM-GRA8-M/AS considerably enhanced the association of rGRA8 with ATP5A1, SIRT3, and ATP5C1. Furthermore, the deacetylation of ATP5A1 and mitochondrial protein synthesis increased in tumor lysates by IB (Figure 4A, bottom). Remarkably, rATRAM-GRA8-M/AS had an IC_50_ of 20 μg/kg that was a 500-fold improvement in IC_50_ compared with that of 10 mg/kg for rGRA8 alone *in vivo* (Figure 4A and Supplementary Figure 2B).

To clarify the roles of PKCα, SIRT3, and ATP5A1 in rATRAM-GRA8-M/AS-induced antitumor activity, we generated PKCα-, SIRT3-, and ATP5A1-deficient HCT116 cells using specific lentiviral shRNAs. We then assessed the tumor-suppressive effects of shPKCα, shSIRT3, and shATP5A1-HCT116 cells in xenograft mice treated with rATRAM-GRA8-M/AS. Reduction in tumor size and delay in tumor growth by rATRAM-GRA8-M/AS was dependent on PKCα, SIRT3, ATP5A1 (Figure 4C–4E and Supplementary Figure 3A), and the acetylation status of ATPA1 K506 and K531 (Figure 4F and Supplementary Figure 3B) in the HCT116 colon-cancer xenograft model.

An important factor in developing therapeutic proteins is immunogenicity because of both safety and efficacy are closely linked with it [24]. We therefore assessed the immunogenicity of rATRAM-GRA8-M/AS by measuring the generation of anti-rATRAM-GRA8-M/AS antibodies in mice immunized with this peptide. After three successive injections with rATRAM-GRA8-M/AS, the production of IgG antibodies was determined using ELISA (enzyme-linked immunosorbent assay). Ovalbumin was employed as a positive control. There was negligible production of specific anti-rATRAM-GRA8-M/AS antibodies, as compared to ovalbumin induced production of antibodies (Supplementary Figure 3C). In addition, proinflammatory cytokine production induced by rATRAM-GRA8-M/AS was negligible in mice with tumors. There was no difference between the negative control and rATRAM-GRA8-M/AS administered mice in the serum levels of IL-1β, TNF-α and IL-6, (Supplementary Figure 3D), indicating that rATRAM-GRA8-M/AS triggers insignificant immunogenicity and immune responses in BALB/c mice. Thus, rATRAM-GRA8-M/AS is a biocompatible biomaterial and rATRAM-GRA8-M/AS-mediated mitochondrial metabolic resuscitation is crucial for its antitumor activity.

## DISCUSSION

The present study found a novel therapeutic approach for cancer on the basis of rATRAM-GRA8-M/AS-mediated mitochondrial metabolic resuscitation. This represents a major paradigm shift in cancer therapy and an urgently needed therapeutic intervention. Notably [1], design and expression of ATRAM-conjugated multifunctional GRA8 peptide to target tumor cells *in vitro* and *in vivo* [2], rATRAM-GRA8-M/AS induced HCT116 cell death by mitochondrial activation [3], rATRAM-GRA8-M/AS showed antitumor activity in HCT116 xenografts. We found that the ATRAM-conjugated multifunctional GRA8 peptide might be exploit in therapeutic interventions against tumors. Furthermore, a proof-of-concept was provided by our work, for therapeutic strategies that are host-directed and that manipulated GRA8-induced host metabolic networks in cancer.

There is an increase in research focused on CTPs. ATRAM provides a method for providing therapeutic cargoes and a stronger binding with cancer cells in acidic environment. This underscores its superior cell-targeting capabilities compared with both pH (low)-insertion peptides and proteins with the cytotoxic TAT-conjugated oxygen-dependent degradation domain of HIF-1α under hypoxic conditions of the tumor microenvironment [20, 21, 25].

The rATRAM-GRA8-M/AS peptide has prospective therapeutic uses; however, it does not have the necessary requisites of antitumor agents as a viable alternative to conventional chemotherapy. Many of the protein and peptide-based therapeutics have drawbacks such as reduced bioavailability and higher metabolic lability. Moreover, most of these biologicals have relatively large molecular sizes and many side-groups that are charged and ionic, making these molecules to be less lipophilic, and hindering their absorption [26, 27]. Intraperitoneal administration of these peptide therapeutics bypasses the problem with absorption, even though factors limiting their bioavailability remain. These limiting factors include fast metabolism, subunit dissociation, conformational changes, systemic proteolytic enzymes, opsonization, non-covalent complex formation with blood products, and modification of labile and reactive side-groups [18, 28].

Oral administration increases patient compliance; because of this, there is significant interest in devising systems that permit the protein and peptide therapeutics oral delivery [29]. Further analyses are required to determine noninvasive delivery methods and suitability of rATRAM-GRA8-M/AS designed to bypass the issues related to therapeutic targeting and systemic lability for use in patients with colon cancer.

Peptide-based biologicals have significant potential as immunotherapeutic agents against several malignant cancers. Many studies [30–33] have approached the peptide-based cancer therapeutics development and their delivery by mirroring the corresponding functional side-groups and domains of proteins having highly specific functions as immuno-regulators. Cancer immunotherapy using peptide-based therapeutics likely overcome the present problems of reaching only low levels inside the tumors and also the off-target effects in relation with the use of soluble checkpoint-blocking antibody. Besides, a clear knowledge of the systemic interactions among various peptides and immune systems is presently lacking. Furthermore, one should keep in mind that in the case of human use, the relevant mechanism and the efficiency of the increased permeability and retention are heterogeneous and still controversial [34–36]. The present work not only focusses in comprehensive manner on the categories, design and applications of peptide-based therapeutics for cancer, but also elaborates on the importance of peptides in the regulation of mitochondrial functions. Tumor-targeted peptide-immunotherapeutics potentially have many ‘plus points’ and these include the following: they are economically beneficial as they reduce the cancer burden effectively; it is feasible for manufacturing these peptide therapeutics in clinical-grade, in accordance with ‘good manufacturing practices’; and these peptide therapeutics are suitable for the treatment of solid tumors. Peptide-based immune therapeutics can be better employed with significant clinical breakthroughs, by exploiting the effects of amino acid chain length of the peptides and their formulations on the peptide immunogenicity. Moreover, there are certain important challenges that remain to be addressed to improve the next-generation development of peptide immunotherapeutics. These include: adapting the peptide synthetic procedures for large-scale production, safe and effective target-oriented delivery, and effective immunotherapy of cancers. In conclusion, in order to effectively maximize the anti-cancer effectiveness of immunotherapies, it is important to combine their efficacy for tumor-targeting as well as specificity for tumor immune cell type. The prospect of treating cancers using the next-generation peptide-immunotherapeutics will be dependent on the results of translational and clinical trials in future.

## MATERIALS AND METHODS

### Ethics statement

All animal experimental procedures were reviewed and approved by the Institutional Animal Care and Use Committee (protocol 2016-0221 and 2017-0218) and by the Institutional Review Board (HYI-17-227-1) of Hanyang University. All animal experiments were performed in accordance with the Korean Food and Drug Administration guidelines.

### Mice and cell culture

Wild-type C57BL/6 and BALB/c mice were purchased from Orient Bio (Gyeonggi-do, Korea). Primary bone marrow–derived macrophages (BMDMs) were isolated from C57BL/6 mice and cultured in DMEM for 3–5 d in the presence of M-CSF (R&D Systems, 416-ML), as described previously [19]. HEK293T cells (ATCC-11268; American Type Culture Collection), human colon cancer cell line HCT116 (ATCC-CCL247), HT-29 (ATCC-HTB-38), HepG2 (ATCC-HB-8065), Hep3B (ATCC-HB-8064), MCF7 (ATCC-HTB-22), and MDA-MB-231 (ATCC-HTB-26) were maintained in DMEM (Invitrogen) containing 10% FBS (Invitrogen), sodium pyruvate, nonessential amino acids, penicillin G (100 IU/ml), and streptomycin (100 μg/ml). Transient transfections were performed using Lipofectamine 3000 (Invitrogen) in HCT116, or calcium phosphate (Clontech) in 293T, according to the manufacturer’s instructions. HCT116 stable cell lines were generated using a standard selection protocol with 400–800 μg/ml of G418.

### Recombinant ATRAM-GRA8 Mito-ATP5A1/SIRT3 protein

To obtain recombinant purified protein, ATRAM amino acid residues (GLAGLAGLLGLEGLLGLPLGLLEGLWLGLELEGN), mitochondria targeting seq. (ATATTTTTTR), and ATP5A1/SIRT3 binding seq. (YRFTVQTTQN) were cloned with an N-terminal 6xHis tag into the pRSFDuet-1 Vector (Novagen) and induced, harvested, and purified from *Escherichia coli* expression strain BL21(DE3) pLysS as described previously [8, 19], in accordance with the standard protocols recommended by Novagen. rGRA8 was dialyzed with permeable cellulose membrane and tested for lipopolysaccharide contamination with a *Limulus* amebocyte lysate assay (BioWhittaker) and contained < 20 pg/ml at the concentrations of rGRA7 protein used in the experiments described here.

### Reagents and antibodies

The target shRNA plasmid DNA human PKCα (RHS4531-EG5578) and SIRT3 (RHS4531-EG23410) were purchased from Open Biosystems. Specific antibodies against ATP5C1 (PA5-29975) was purchased from Invitrogen. Abs specific for ATP5A1 (51), SIRT3 (14.45), VDAC (B-6), SDHA (B-1), UQCRC2 (G-10), COX IV (D-20), PGC-1β (E-9), Tfam (H-203), PKCα (C-20), Actin (I-19), V5 (H-9), Flag (D-8), His (AD1.1.10), Lys-AC (AKL5C1), and GST (B-14) were purchased from Santa Cruz Biotechnology.

### Plasmid construction

The plasmid encoding full-length of the GRA8, ATP5A1, and SIRT3 plasmid were previously described [8]. The list of the plasmids in this study are described in Table 3.

**Table 3:** Lists of plasmid used in the study

Name	Expression	Tag or Clon ID	References
pET23b-Vector	Bacterial expression	His	[8]
pET23b-GRA8 WT (1-269)	Bacterial expression	His	[8]
pET23b-GRA8 T220A	Bacterial expression	His	[8]
pET23b-GRA8 N (1-241)	Bacterial expression	His	[8]
pEBG-GRA8 WT (1-269)	Mammalian expression	GST	[8]
pcDNA3-ATP5A1	Mammalian expression	V5	[8]
pcDNA3- ATP5A1 (K506, 531R)	Mammalian expression	V5	[8]
pcDNA3-SIRT3	Mammalian expression	Flag	[8]
pET23b-ATRAM-G8-M/AS	Bacterial expression	His	In this study
pET23b-ATRAM-G8-M	Bacterial expression	His	In this study
pET23b-ATRAM	Bacterial expression	His	In this study
pGIPZ-shPKCα	Human	V2LHS_218226, V2LHS_87125, V2LHS_170435, V2LHS_170433 From Horizon Discovery Dharmacon	In this study
pGIPZ-shSIRT3	Human	V3LHS_365648, V3LHS_347987, V3LHS_365650 From Horizon Discovery Dharmacon	In this study
pGIPZ-shATP5A1	Human	V2LHS_192755, V2LHS_218429, V3LHS_375667, V3LHS_375664 From Horizon Discovery Dharmacon	In this study

### Peptides

Tat-conjugated GRA8 peptides were commercially synthesized and purified in acetate salt form to avoid abnormal responses in cell by Peptron (Korea). The amino acid sequences of the peptides in this study are described in Table 2. The endotoxin content, as measured by the Limulus amebocyte lysate assay (BioWhittaker) and contained less than 3–5 pg/ml at the concentrations of the peptides used in experiments.

### GST pulldown, immunoblot, and immunoprecipitation analysis

GST pulldown, immunoprecipitation, and immunoblot assays were performed as described previously [8, 19]. For GST pulldown, cells were harvested and lysed in NP-40 buffer supplemented with a complete protease inhibitor cocktail (Roche). After centrifugation, the supernatants were precleared with protein A/G beads at 4°C for 2 h. Pre-cleared lysates were mixed with a 50% slurry of glutathione-conjugated Sepharose beads (Amersham Biosciences), and the binding reaction was incubated for 4 h at 4°C. Precipitates were washed extensively with lysis buffer. Proteins bound to glutathione beads were eluted with SDS loading buffer by boiling for 5 min.

For immunoprecipitation, cells were harvested and then lysed in NP-40 buffer supplemented with a complete protease inhibitor cocktail (Roche). After pre-clearing with protein A/G agarose beads for 1 h at 4°C, whole-cell lysates were used for immunoprecipitation with the indicated antibodies. Generally, 1–4 μg of commercial antibody was added to 1 ml of cell lysates and incubated at 4°C for 8 to 12 h. After the addition of protein A/G agarose beads for 6 h, immunoprecipitates were extensively washed with lysis buffer and eluted with SDS loading buffer by boiling for 5 min.

For immunoblotting, polypeptides were resolved by SDS-polyacrylamide gel electrophoresis (PAGE) and transferred to a PVDF membrane (Bio-Rad). Immuno detection was achieved with specific antibodies. Antibody binding was visualized by chemiluminescence (ECL; Millipore) and detected by a Vilber chemiluminescence analyzer (Fusion SL 3; Vilber Lourmat).

### Cellular fractionation

Cytosol and mitochondria were isolated from cells using a Mitochondria Fractionation Kit (Active Motif, 40015) or as described previously [8]. Subcellular fractionated proteins were lysed in buffer containing 2% SDS and boiled with 2× reducing sample buffer for SDS-PAGE.

### MTT assay

Cell viability relative to non-treated group was measured by MTT assay, as described previously [8]. After incubating for the indicated time points, 5 mg/ml of MTT (3-(4,5-dimethylthiazol-2-yl)-2,5-diphenyltetrazolium bromide) solution was added in the place of media, and cells were incubated for further 4 h. Then, all the media was removed and the same volume of dimethyl sulfoxide (DMSO) solution was added for 15 min to dissolve the formazan. Using UV/VIS spectrophotometer, each well of the plate was measured at 540 nm to measure relative cell viability.

### Mitochondrial DNA quantification

To quantify mtDNA copy number, we measured the mitochondrial (mt) to nuclear (n) DNA ratio, as described previously [37]. Pyruvate kinase (*Pklr*) was used as a marker for nDNA and NADH dehydrogenase subunit 1 (*mt-Nd1*) for mtDNA. Real-time PCR reactions were performed according to the manufacturer’s instructions (QuantiFast SYBR green PCR master mix; Qiagen, 204052), and thermal cycling was performed in a QuantStudio™ 3 (ABI). The mtDNA content was normalized to the nucleic DNA content.

### Mitochondrial membrane potential measurements

The mitochondrial membrane potential (ΔΨm) of intact cells was measured as described previously [38] with modifications. TMRE (tetramethylrhodamine, ethyl ester; 200 nM, Molecular Probes-Invitrogen, T669) was added to the cell suspension. Cells were incubated at 37°C for 30 min in the dark. ΔΨm was measured by flow cytometry, and data were analyzed using the FlowJo software. TMRE fluorescence was measured using the FL2 channel (582 nm).

### Complex V activity assay

The activity of complex V was determined using the MitoTox Complex V OXPHOS Activity Microplate Assay kit from Abcam (ab109907, Cambridge, MA, USA), as described previously [38]. The activity of complex V was measured by monitoring the change in absorbance at 340 nm over a period of 1 hour at 30°C. Oligomycin (Sigma, O4876) was used as a positive control for the assay.

### Mouse model xenograft

Female athymic nude mice 4 to 6 weeks in age (Central Lab. Animal, Korea) were used for the tumor xenograft experiments, as previously described [37]. Briefly, HCT116 is a KRAS proto-oncogene mutant, used in colon cancer tumorigenicity screening studies and metastasis model. HCT116 is positive for transforming growth factor beta (TGFβ) 1 and TGFβ2 expression [39]. HCT116 cells were harvested from the culture flask and suspended in culture medium. Animals were injected with 1 × 10^6^ HCT116 cells suspended in 0.1 mL cell medium subcutaneously into the right axillary region and observed for 7–10 days with a tumor volume measurement. The treatment was initiated when the tumor size reached an average volume of 200 or 600 mm^3^. The tumor volume was measured every third day with skin calipers and calculated as the tumor length × tumor width^2^ × 0.5, and represented in mm^3^. All animals were maintained in a specific pathogen-free environment.

### Statistical analysis

All data were analyzed using Student’s *t*-test with Bonferroni adjustment or ANOVA for multiple comparisons, and are presented as mean ± SD. Statistical analyses were conducted using the SPSS (Version 12.0) statistical software program (SPSS). Differences were considered significant at *p* < 0.05.

## SUPPLEMENTARY MATERIALS


