# Supplementary material for: Toxoplasma gondii GRA8-derived peptide immunotherapy improves tumor targeting of colorectal cancer
Source: Oncotarget. 2020 Jan 7;11(1):62–73. doi: 10.18632/oncotarget.27417 (PMC6967779; doi:10.18632/oncotarget.27417)
Supplement: Supplementary file 1 [file oncotarget-11-62-s001.pdf]

Toxoplasma gondii GRA8-derived peptide immunotherapy improves tumor targeting of colorectal cancer

SUPPLEMENTARY MATERIALS

Methods

IgG antibody responses

For immunization, BALB/C mice were injected with rATRAM-GRA8-M/AS (20 µg/kg), ovalbumin (10 mg/kg), or PBS on every 3 day. At 40 days, after the final immunization, blood were collected to assess the serum IgG. The level of immunoglobulin G (IgG) antibody in the mice sera was analyzed as described elsewhere [1]. Briefly, sera from the mice were diluted at 1:320 in PBST, and 100 µl of the diluted samples was added to the anti-mouse IgG (1 µg/ml) coated wells. The optical density at 450 nm was measured using an ELISA plate reader (Bio-Rad, Hercules, CA, USA).

Enzyme-linked immunosorbent assay

Mice sera were analyzed using BD OptEIA ELISA Kit (BD Biosciences, Franklin Lakes, NJ, USA) for the detection of TNF-α, IL-6, and IL-1β, as recommended by the manufacturers [2].

REFERENCES

1. Lee JJ, Kim HJ, Yang CS, Kyeong HH, Choi JM, Hwang DE, Yuk JM, Park K, Kim YJ, Lee SG, Kim D, Jo EK, Cheong HK, et al. A high-affinity protein binder that blocks the IL-6/STAT3 signaling pathway effectively suppresses non-small cell lung cancer. Mol Ther. 2014; 22:1254–65. https://doi.org/10.1038/mt.2014.59. [PubMed]

2. Koh HJ, Kim YR, Kim JS, Yun JS, Jang K, Yang CS. Toxoplasma gondii GRA7-Targeted ASC and PLD1 Promote Antibacterial Host Defense via PKCalpha. PLoS Pathog. 2017; 13:e1006126. https://doi.org/10.1371/journal.ppat.1006126. [PubMed]

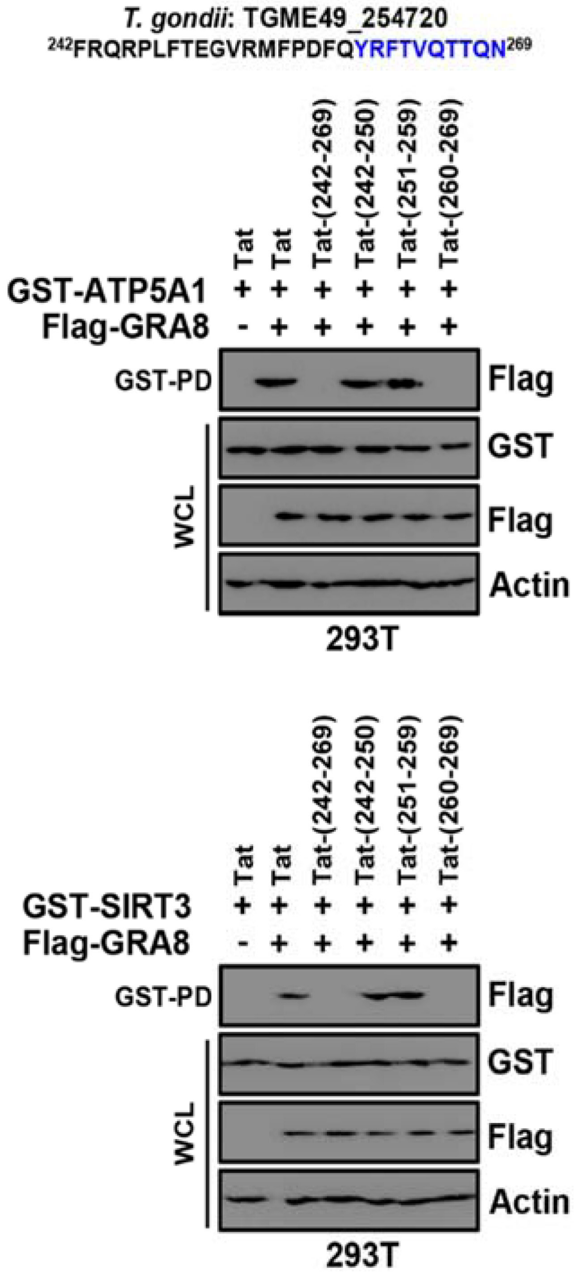

Supplementary Figure 1: Identified minimal region of GRA8 directly interacts with ATP5A1 and SIRT3. At 12 hr post-transfection with mammalian GST-ATP5A1 or GST-SIRT3 constructs together with Flag-GRA8 and 293T cells treated with several Tat-GRA8 peptide (10 μM) for 6 h. 293T cells were used for GST pulldown, followed by IB with αFlag. WCLs were used for IB with αGST, αFlag or αActin. The data are representative of four independent experiments with similar results.

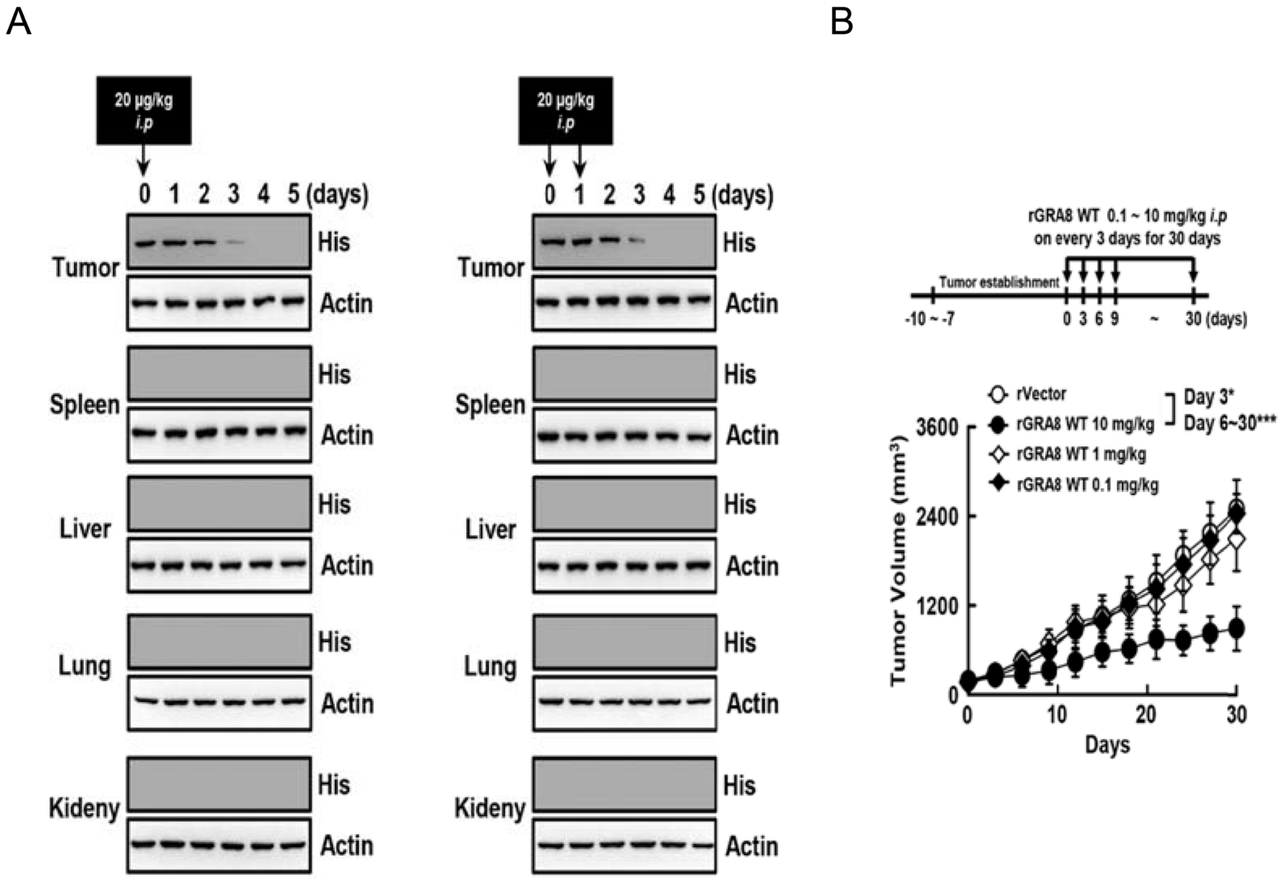

Supplementary Figure 2: The pharmacokinetics and pharmacodistribution of rATRAM-GRA8-M/AS. (**A**) The pharmacokinetics and pharmacodistribution against rATRAM-GRA8-M/AS in tumor-bearing mice. HCT116 cells were subcutaneously injected into the flanks of BALB/c mice, as described in the Methods section. The mice were administrated by i.p. at one or two times and sacrificed on day 5. His expression was assessed by immunoblotting (IB) in various organs from mice. Whole-cell lysates were used for the IB with αActin. The data are representative of four independent experiments with similar results. (**B**) Schematic of the xenograft model treated with or rGRA8 WT (upper). HCT116 cells were subcutaneously injected into the flanks of nude mice. The length and width of the tumors were measured using calipers, and the tumor volume was calculated every third day for 30 days. Individual tumor volumes from each mouse from each group were averaged and plotted against the number days postinoculation. Statistical significance was determined by two-way analysis of variance (ANOVA) with Tukey's posttest; $^{*}P < 0.05$, $^{***}P < 0.001$ compared with rVector. Each group contained ten mice. The data are representative of two independent experiments with similar results.

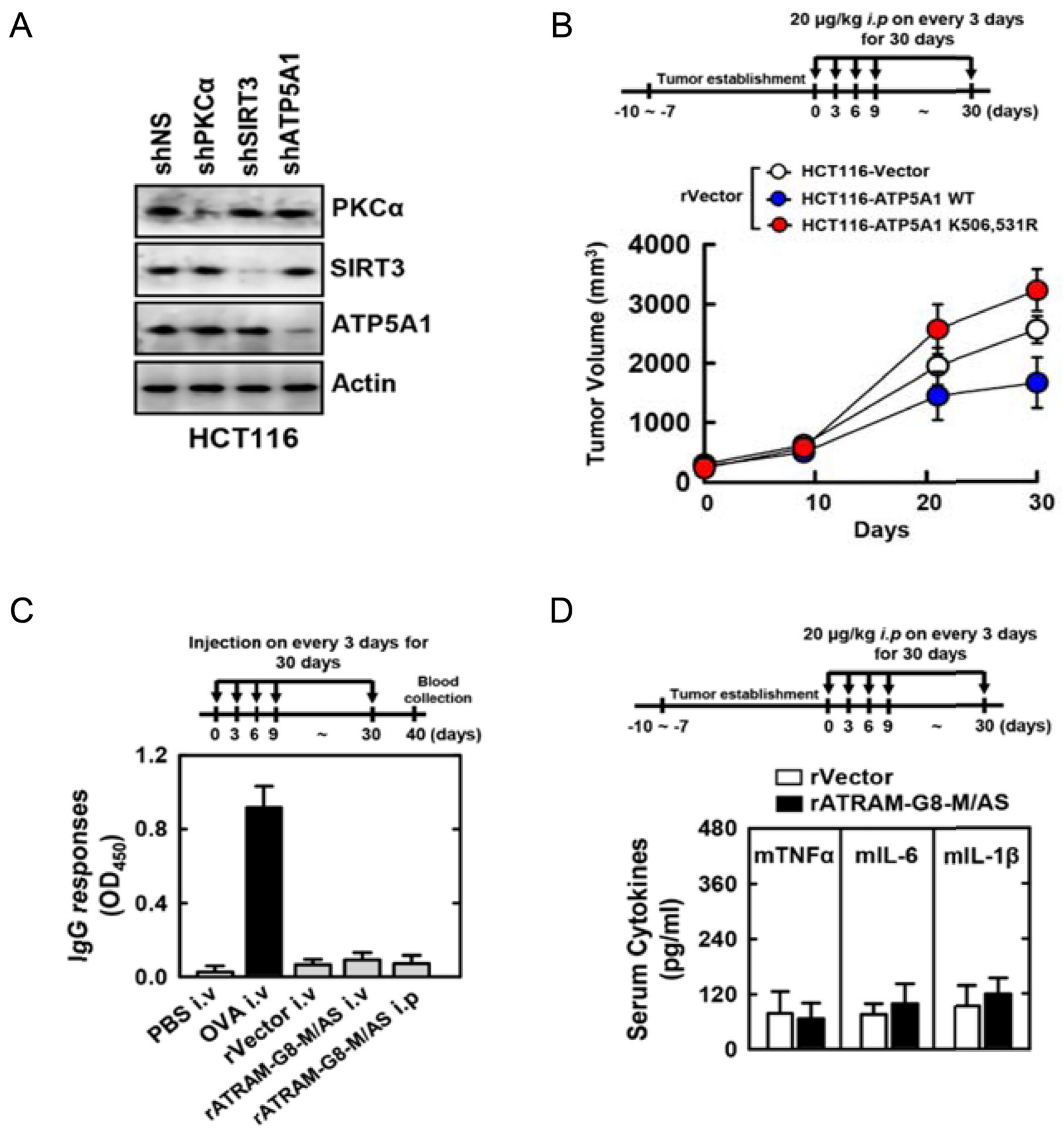

Supplementary Figure 3: The knock-down effects in HCT 116 and immunogenicity of rATRAM-GRA8-M/AS *in vivo*. (**A**) HCT116 cells were transduced with lentivirus-shRNA-NS or lentivirus-shRNA-PKCα, SIRT3, or ATP5A1 with polybrene (8 μg/mL) for 2 days. IB with αPKCα, αATP5A1, αSIRT3, or αActin. The data are representative of five independent experiments with similar results. (**B**) Schematic of the xenograft model treated with rVector (upper). HCT116-expressed ATP5A1 WT and mutant cells were subcutaneously injected into the flanks of nude mice. Significant differences compared with rVector. Each group contained ten mice. The data are representative of two independent experiments with similar results. (**C**) Humoral immune responses in BALB/C mice. The mice were immunized at each route (i.v. or i.p.) on every 3 day and bled on day 40. The level of IgG specific for rATRAM-GRA8-M/AS or ovalbumin (OVA) was measured using ELISA. The values indicate the means of optical density (OD) and standard deviation at 450 nm. Each group contained five mice. (**D**) Cytokine responses against rATRAM-GRA8-M/AS in tumor-bearing mice. HCT116 cells were subcutaneously injected into the flanks of BALB/c mice, as described in the Methods section. The serum cytokine levels were determined at 30 days after administration. Each group contained ten mice.
